# Does vitamin D deficiency contribute to post-burn bone loss?

**DOI:** 10.12688/f1000research.1-57.v1

**Published:** 2012-11-28

**Authors:** Gordon L Klein

**Affiliations:** 1 Department of Orthopaedic Surgery and Rehabilitation and Shriners Burns Hospital, University of Texas Medical Branch at Galveston, Galveston, 77555 TX, USA

## Abstract

Burn injury results in the acute loss of bone as well as the development of progressive vitamin D deficiency. Bone loss occurs acutely due to resorption, which is then followed by apoptosis of osteoblasts preventing repair of the bone loss. The acute resorption is due to a combination of the inflammatory response and the stress response to the burn injury. The resultant production of inflammatory cytokines and endogenous glucocorticoids initially stimulate the osteoblasts to produce RANK ligand, which stimulates marrow stem cell differentiation into osteoclasts. As the stress response persists for approximately one year post-burn the glucocorticoids produced by the body will cause osteoblast apoptosis and adynamic bone, impairing the ability of bone to recover its resorptive losses. The vitamin D deficiency is due to the failure to supplement the diet of burn patients with vitamin D on discharge from hospital and to failure of the skin to make normal quantities of vitamin D on sunlight exposure. Because the bone resorption can be prevented by the acute administration of bisphosphonates it is unlikely that vitamin D deficiency is responsible for the early-onset bone loss following burns. However, because a deficit in trabecular bone remains for at least two years post-burn, it is possible that vitamin D deficiency prevents the recovery of trabecular bone density over the long term.

## Commentary

Children who have been severely burned develop a progressive vitamin D deficiency ^1^ as characterized by low circulating levels of 25-hydroxyvitamin D (25(OH)D) between 14 ^2^ and 24 months post-burn with normal circulating levels of 1,25-dihydroxyvitamin D (1,25(OH) _2_D). Unlike rickets there is no compensatory rise in 1,25(OH) _2_D and this may be due to persistent low serum parathyroid hormone (PTH) concentrations ^2, 3^. The hypoparathyroidism is likely due to a persistent post-burn up-regulation of the parathyroid calcium sensing receptor ^3, 4^. By 7 years post-burn, there is a continued reduction in serum levels of 25(OH)D in all patients but now there is also a reduction in circulating concentrations of 1,25(OH) _2_D ^1^.

There are at least two reasons for this progressive deficiency. The first explanation is that burn patients are not routinely given vitamin D supplements following hospital discharge ^1^ and even if they are given a standard amount of vitamin D supplementation, i.e. 400 international units per day, for six months post-burn, serum levels of 25(OH)D are found to be in the "insufficient" range, approximately 20 ng/ml ^5^. Therefore, the proper dose of vitamin D to administer to these children is unknown. The second explanation is that following burn injury, not only the burn scar tissue but also the adjacent normal-appearing skin cannot convert normal quantities of the precursor 7-dehydrocholesterol (7DHC) to vitamin D _3_ when subjected to UVB irradiation ^2^. Moreover, the epidermal cells both in burn scar tissue and in adjacent normal-appearing skin contain subnormal quantities of 7DHC suggesting abnormalities in cholesterol biosynthesis in skin following burn injury ^2^.

Bone loss following burn injury can be attributed to least two non-specific adaptive responses: the inflammatory response and the stress response. Both occur as a result of the destruction of skin, which is an important barrier to infection. The inflammatory response involves significant elevations of circulating cytokines, especially interleukin (IL)-1β and IL-6 ^6^. The stress response is characterized by a 3–8-fold rise in endogenous glucocorticoid production as measured by urine free cortisol excretion ^6, 7^. Acutely, both processes stimulate osteoblast production of the ligand of the receptor activator of NFκB (RANKL), which then stimulates marrow stem cell differentiation into osteoclasts and results in increased bone resorption. The success of acute intravenous administration of bisphosphonates either as a single or once-repeated dose in preventing the bone loss ^8^ is evidence that this is the case. Because the stress response is sustained for as long as one year post-burn, persistence of the elevated endogenous production of glucocorticoids leads to osteoblast and likely osteocyte apoptosis and reduction of marrow stromal cell differentiation into osteoblasts ^7^. Thus the now adynamic bone cannot recover the deficit caused by the acute resorption. It is only after the stress response has dissipated after about one year post-burn, that remodeling resumes ^9^.

Because the intravenous administration of bisphosphonates prevents the acute bone loss entirely ^8^ for up to two years ^9^, it is highly unlikely that the documented vitamin D deficiency plays a role in the immediate post-burn bone loss. Moreover, we have also shown that, at least acutely, there are low circulating levels of both vitamin D binding protein and albumin ^10^, with albumin recovering by 6 months post-burn ^5^. Therefore, whether there is true vitamin D deficiency during the first six months post-burn is difficult to determine. Nevertheless, what we do see in these burn patients is that by two years post-burn, cortical bone deficits, as measured by total body bone mineral content, in those not receiving acute bisphosphonate therapy recover to the level of those who did receive bisphosphosphonates ^9^ while the deficit in trabecular bone, as measured by lumbar spine bone density, persisted ^9, 11^.

A recent report by Zhou *et al.* ^12^ has shown that in adult human marrow stromal cell culture, the clinical conditions under which the bone biopsies were obtained influenced the ability of the stromal cells to differentiate into osteoblasts in response to 1,25(OH) _2_D. In particular it was noted that vitamin D deficiency impaired this differentiation. This finding raises the possibility that progressive vitamin D deficiency as experienced by children post-burn may explain the persistence of their trabecular bone deficit, although why vitamin D deficiency would uniquely affect trabecular bone is not at all clear. Perhaps future studies looking at the differential effects of vitamin D on cortical and trabecular bone might determine whether this hypothesis is viable.
