# Supplementary material for: Does vitamin D deficiency contribute to post-burn bone loss?
Source: F1000Res. 2012 Nov 28;1:57. [Version 1] doi: 10.12688/f1000research.1-57.v1 (PMC3752641; doi:10.12688/f1000research.1-57.v1)
Supplement: Peer review history

# Peer review: Referee response for version 1

**Published:** 2013-01-22

**Authors:** Jon Tobias

**Affiliations:** Musculoskeletal Research Unit, Bristol Royal Infirmary, Bristol, UK

My reservations are as follows:

1. Since children are not routinely supplemented with vitamin D, the lack of vitamin D supplementation in burn victims is not in itself an explanation for their low vitamin D levels.

2. The assertion that elevated endogenous glucocorticoids contribute to bone loss in this context, through inhibition of osteoblast survival and generation, is somewhat over-stated. Although adverse effects of glucocorticoids on osteoblast function are well described, the literature mainly applies to pharmacological doses of glucocorticoid. The evidence cited here, concerning possible adverse effects of endogenous glucocorticoids, relates to a small observational study which provides weak circumstantial evidence at best.

I have read this submission. I believe that I have an appropriate level of expertise to confirm that it is of an acceptable scientific standard, however I have significant reservations, as outlined above.

# Response

**Published:** 2012-11-30

**Authors:** Gordon Klein

**Affiliations:** Department of Orthopaedic Surgery and Rehabilitation and Shriners Burns Hospital, Unievrsity of Texas, Medical Branch, USA

The comments from Dr Tobias are greatly appreciated. If I did not make it clear in the commentary I listed two contributory factors to post-burn vitamin D deficiency. The failure to supplement is not of itself a causative factor, I agree, but the fact that the skin cannot make vitamin D normally on exposure to UVB radiation coupled with the lack of routine supplementation means that the burn victims have no way to compensate for the skin’s failure to normally synthesize vitamin D3 from its precursor.

With regard to the comment on endogenous glucocorticoids clearly Dr Tobias is correct in stating that the literature on glucocorticoid-induced osteoporosis relates to the exogenous administration of glucocorticoids. the studies that I have cited, albeit they are my own, demonstrate that the body produces large quantities of endogenous glucocorticoids in response to stress, coupled with lack of surface osteoblasts and a reduction in markers of osteoblast differentiation in marrow stromal cells taken from the burn victims as compared to unburned controls. The point to be emphasized, I believe, is that currently there is no way to equate the quantity of endogenous glucocorticoids produced in response to stress with the dosage of glucocorticoids given as therapy. In the absence of work corroborating our observations I raise the issue of similar effects of endogenous and exogenous glucocorticoids in order to increase awareness that in the case of burns at least the classic manifestations of glucocorticoid induced osteoporosis also exist in children who did not receive exogenous glucocorticoids, I would certainly agree that more studies in this area and in different conditions in which a patient is subject to stress would help clarify the situation, but I would not dismiss the argument for lack of evidence.

# Peer review: Referee response for version 1

**Published:** 2012-11-30

**Authors:** Daniel Bikle

**Affiliations:** Department of Medicine and Dermatology, University of California/VAMC, San Francisco, CA, USA

I have read this submission. I believe that I have an appropriate level of expertise to confirm that it is of an acceptable scientific standard.

# Peer review: Referee response for version 1

**Published:** 2012-11-28

**Authors:** Dominique Garrel

**Affiliations:** Endocrinology Department, Hotel Dieu de CHUM Hospital, Montreal, Canada

I have read this submission. I believe that I have an appropriate level of expertise to confirm that it is of an acceptable scientific standard.
